# Impact of severe aortic stenosis on quality of life

**DOI:** 10.1371/journal.pone.0287508

**Published:** 2023-06-21

**Authors:** Juan Manuel Gracia Baena, Josep Ramon Marsal Mora, Sara Llorca Cardeñosa, Imma Calaf Vall, Marta Zielonka, Pere Godoy

**Affiliations:** 1 Cardiac Surgery Department, Vall d’Hebron University Hospital, Barcelona, Spain; 2 Applied Epidemiology Unit, Department of Medicine and Surgery, University of Lleida, Lleida, Spain; 3 Lleida Research Support Unit, Foundation University Institute for Primary Health Care Research Jordi Gol i Gurina (IDIAPJGol), Barcelona, Spain; 4 CIBER Epidemiology and Public Health CIBERESP, Institute of Health Carlos III, Madrid, Spain; 5 Catalan Public Health Agency (ASPCAT), Barcelona, Spain; Universitaria di Bologna, ITALY

## Abstract

**Introduction:**

Among individuals ≥ 65 years old, aortic stenosis is highly prevalent and the number of cases is expected to increase in the coming decades, due to the increased life expectancy. Nevertheless, the actual aortic stenosis burden is not well known in population settings and the impact of aortic stenosis on quality of life has not been studied. The aim of this study was to evaluate aortic stenosis impact on health-related quality of life in patients > 65 years old.

**Methods:**

An epidemiological case-control study was carried out to compare quality of life in patients ≥65 years old with severe symptomatic aortic stenosis. Demographical and clinical information was prospectively obtained and quality of life information was collected with the Short Form Health Survey_v2 (SF-12) questionnaire. The association between quality of life and aortic stenosis was determined using multiple logistic regression models.

**Results:**

Patients with severe aortic stenosis self-perceived worse quality of life on all dimensions and summary components of the SF-12 questionnaire. In the final multiple logistic regression model a significant inverse association was observed between the dimensions ‘physical role’ and ‘social role’ (p = 0.002 and p = 0.005) and an association close to significance with ‘physical role’ (p = 0.052) of the SF-12 questionnaire.

**Conclusion:**

The use of quality of life scales allows the assessment of the impact of aortic stenosis on quality of life and may improve the therapeutic approach to severe aortic stenosis, providing evidence for patient‐centered care.

## Introduction

Aortic stenosis (AS) is the most common surgically managed valvular heart disease and the third most common cardiovascular disease in Western countries [1]. In our regional setting, prevalence in individuals ≥ 65 years is 3%, but increases exponentially with age to reach 7.4% in people > 85 years [2]. As life expectancy rises, it is estimated that case numbers in people > 70 years of age will double or triple in the coming decades [3–5].

Severe AS, if untreated, is associated with high mortality [4]. In the absence of useful pharmacological treatments, the only effective treatments are aortic valve replacement (AVR) or transcatheter aortic valve implantation (TAVI) [6, 7]. The therapeutic decision should take into account the clinical characteristics of the patient, the feasibility of the intervention, local experience and outcomes, and the individualized surgical risk and likelihood that the intervention will improve survival and health-related quality of life (HRQoL) [8, 9].

Quality of life (QoL) can be defined as a multidimensional element that includes physical, psychological, cognitive, and social factors that affect how a person performs their activities and self-perceived degree of well-being [10–12]. QoL assessments monitor the impact of a disease (and/or treatment) on a patient’s personal and social performance and are widely used in clinical practice, and SF-12 is one of the most widely used instruments [13]. This questionnaire provides a generic measurement of general health and well-being and summarizes physical and mental components in 12 items divided into 8 health domains [14–16].

Knowledge of the impact of AS on patients’ QoL in the population setting is still scant. Few studies evaluate QoL in patients with symptomatic severe AS, and these are mostly limited to assessing QoL after surgery (AVR or TAVI) or in patients receiving palliative treatment in whom surgical intervention has been ruled out. Only 1 study in the Netherlands reported that QoL is lower in patients with symptomatic AS [17], but no studies have been conducted on the impact of AS on QoL in the Spanish population. Since the onset of AS symptoms is progressive but slow [4], and patients are elderly with associated comorbidities [18], this could lead to overlook the impact of AS on QoL and the need of surgery. As per current guidelines, patient-related QoL should be considered during the decision on the best treatment option [7]. Indeed, assessing patients’ QoL can promote and provide evidence for patient-centered care [19].

The aim of this study was to assess the impact of symptomatic severe AS on QoL in patients > 65 years, compared to a control group in the same primary care setting, taking into account all cardiovascular risk factors (CVRF) and associated comorbidities.

## Materials and methods

An observational epidemiological case-control study was conducted in a province of Spain between February 2014 and April 2018.

Cases had symptomatic severe AS according to the criteria established by European Society of Cardiology guidelines currently available at the time of patients’ recruitment [9, 20], and were > 65 years. Controls, without known AS, were recruited from the same primary care center as the cases and matched by age (± 5 years) and sex, in a 1:3 ratio. The absolute number of years (± 5) for matching is considered to be high, and was taken into account in logistic regression models to neutralize this potential confounding factor.

Clinical and demographic data were prospectively obtained from patient interviews, physical examination, and consultation of medical records. Data collection, storage, and analysis were carried out according to applicable regulations. The patients gave written informed consent and the study was approved by our Hospital Clinical Research Ethics Committee (CREC) (20/2013) and the participating CREC of the Primary Care Research Institute (P16/132). A comprehensive description of study design, inclusion of cases and controls, and sample size determination can be found elsewhere [21].

Study participants completed the SF-12v2 HRQoL questionnaire. It summarizes the physical and mental components for 12 items across 8 health domains: physical functioning (2 items), role limitations due to physical problems (2 items), bodily pain (1 item), general health (1 item), vitality (1 item), social functioning (1 item), role limitations due to emotional problems (2 items), and mental health (2 items). A non-commercial version of this questionnaire was used, under license number QM021812. The questionnaire responses were interpreted using an algorithm that determines a summary assessment for the Physical Component Summary (PCS) and another for the Mental Component Summary (MCS) dimension from the responses for each item.

Continuous variables were characterized using mean and standard deviation, and categorical variables with absolute and relative frequencies. A multiple logistic regression model was constructed to evaluate the QoL in severe AS patients, adjusted for any variables that were significant in the baseline model. Statistical methods were conducted as previously reported [21]. Briefly, a two-step approach was used. First, potential confounding variables (CVRF, comorbidities) were analyzed at a baseline model. All variables with a p-value <0.2 were selected and their input was carried out using forward and backward variable selection algorithms. Next, the model was readjusted testing those factors that remained in the limit area of p <0.1. Final model was adjusted by age, sex and all significantly associated comorbidities and CVRF.

The strength of the association of the 8 dimensions of the SF-12 questionnaire with the dependent variable was studied using the crude odds ratio with its 95% confidence interval (CI). The independent involvement of each dimension of the questionnaire with severe AS was estimated with the adjusted odds ratio (aOR), where the dependent variable was case/control and the independent variables were the results of the QoL test. Finally, the Receiver Operating Characteristic (ROC) curve was calculated to determine the sensitivity of the models created. All analyses were performed using the SPSS statistical package 15.0 and R, and significance level was set at 0.05.

The raw data is in a private repository and could be shared on request in a confidential manner.

## Results

### Patient characteristics

A total of 102 cases and 221 controls were included in this study. For each case, between 1 and 3 controls from the same primary care center were included, matched by sex and age. An average of 2.17 controls was obtained for each case. The mean age of cases was significantly higher than in controls (77.6 years vs. 75.5 years, p = 0.003) (Table 1), so the variable “age” was taken into account in regression models to avoid the confounding effect. No significant differences were found with regard to sex or other anthropometric variables, except in waist circumference and the presence of central obesity (Table 1). A comprehensive description of baseline demographics and clinical characteristics of cases and controls included in the study can be found elsewhere [21].

**Table 1:** Baseline demographical and clinical characteristics of included patients.

	Cases (n = 102)	Controls (n = 221)	P value
Age (years), mean ± SD	77.6 ± 5.4	75.5 ± 6.1	**0.003** *
Gender, n (% female)	39 (38.2%)	92 (41.6%)	0.564
Weight (kg), mean ± SD	77 ± 12.9 ^a^	74.6 ± 12.4 ^b^	0.076
Waist circumference (cm), mean ± SD	107.1 ± 8.6^c^	102.9 ± 11.1 ^d^	**0.003** *
Central obesity^†^, n (%)	45 (83.3%) ^c^	109 (67.3%) ^d^	**0.024** *
Heart rhythm, n (%)			**<0.001** *
Sinus	71 (69.6%)	180 (90%) ^e^	
Fibrillation / Flutter	28 (27.5%)	19 (9.5%) ^e^	
Pacemaker	2 (2%)	0 (0%) ^e^	
Angina pectoris, n (%)			0.325
• CCS 1	75 (73.5%)	180 (83.3%) ^f^	
• CCS 2	20 (19.6%)	19 (8.8%) ^f^	
• CCS 3	7 (6.9%)	14 (6.5%) ^f^	
• CCS 4	0 (0%)	3 (1.4%) ^f^	
Pooled angina pectoris groups, n (%)			**0.050** *
• CCS 1	75 (73.5%)	180 (83.3%) ^f^	
• CCS 2–4	27 (26.5%)	36 (16.7%) ^f^	
NYHA class, n (%)			**<0.001** *
• 1	11 (10.8%)	174 (80.6%)^f^	
• 2	51 (50%)	25 (11.6%) ^f^	
• 3	39 (38.2%)	15 (6.9%) ^f^	
• 4	1 (1%)	2 (0.9%) ^f^	
NYHA (III-IV) class, n (%)	40 (39.2%)	17 (7.7%) ^g^	**<0.001** *

^†^Defined by waist circumference > 102 cm in male and > 88 cm in female.

*p <0.05.

CCS, Canadian Cardiovascular Society; NYHA, New York Heart Association; SD, standard deviation

^a^ n = 100

^b^ n = 206

^c^ n = 54

^d^ n = 162

^e^ n = 200

^f^ n = 216

^g^ n = 220

### Quality of life evaluations

**Fig 1:**
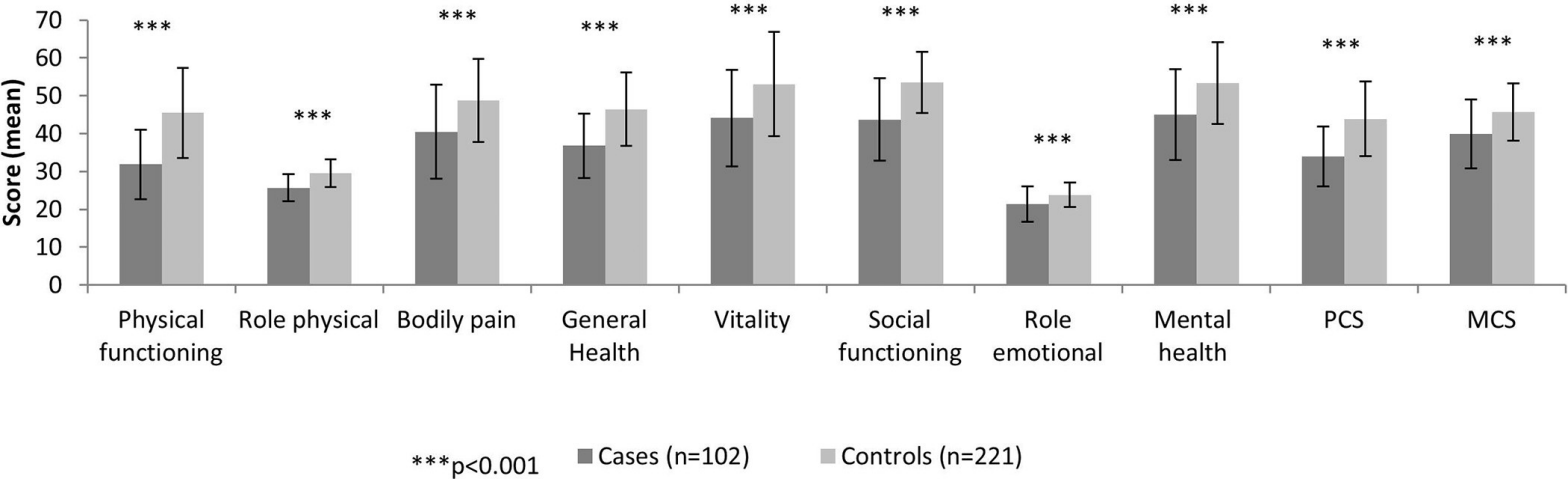
Quality of life score of all dimensions and summary components of the SF-12 questionnaire in symptomatic patients with AS ≥ 65 years old. MCS: Mental Component Summary, PCS: Physical Component Summary. Each bar indicates the score (0–100) for cases and controls of each specific dimension. The bounds are indicating the mean ± standard deviation.

Patients with severe AS had significantly lower QoL compared with healthy controls in all dimensions of the SF-12 questionnaire (p <0.001), and in the PCS and MCS components (p <0.001 for both comparisons) (Fig 1).

Then, the association of symptomatic severe AS with each QoL questionnaire dimension was analyzed. All QoL dimensions were inversely associated with symptomatic severe AS (p <0.001) (data not shown).

CVRFs and comorbidities are associated with the risk of symptomatic severe AS [21]. We explored the association between CVRF and comorbidities and symptomatic severe AS in our data set, and found that the following variables were significantly associated (p <0.05): age, hemoglobin, smoking, hypercholesterolemia, low HDL-cholesterol, stroke, carotid stenosis, and chronic kidney failure.

Next, we adjusted our model for all CVFR and comorbidities significantly associated with severe symptomatic AS. The association between each dimension of QoL and symptomatic severe AS was still significant when adjusted for CVRFs and comorbidities (p <0.001) (Table 2). The area under the ROC curve showed good discrimination and predictive ability of association in all cases (ROC 0.82 to 0.87) (Table 2).

**Table 2:** Logistic regression model adjusted for statistically significant variables in the baseline model (CVRF and comorbidities).

Dimension	aOR (95%CI)^†^	P value	ROC
Physical functioning	0.90 (0.88–0.93)	<0.001*	0.87 (0.83–0.92)
Role physical	0.76 (0.69–0.83)	<0.001*	0.86 (0.82–0.91)
Bodily pain	0.95 (0.92–0.97)	<0.001*	0.83 (0.77–0.87)
General health	0.89 (0.86–0.93)	<0.001*	0.86 (0.82–0.91)
Vitality	0.94 (0.92–0.97)	<0.001*	0.85 (0.80–0.91)
Social functioning	0.91 (0.88–0.94)	<0.001*	0.85 (0.80–0.90)
Role emotional	0.86 (0.80–0.93)	<0.001*	0.82 (0.76–0.87)
Mental health	0.93 (0.91–0.96)	<0.001*	0.854 (0.78–0.89)
PCS	0.89 (0.86–0.93)	**<0.001**	0.71 (0.64–0.77)
MCS	0.90 (0.87–0.94)	**<0.001**	0.71 (0.65–0.78)

aOR, adjusted odds ratio; CI, confidence interval; MCS, Mental Component Summary; PCS, Physical Component Summary; ROC, Receiver operating characteristic.

^**†**^ aOR (age, hemoglobin, smoking, hypercholesterolemia, low HDL-cholesterol, stroke, carotid stenosis, and chronic kidney failure)

**Fig 2:**
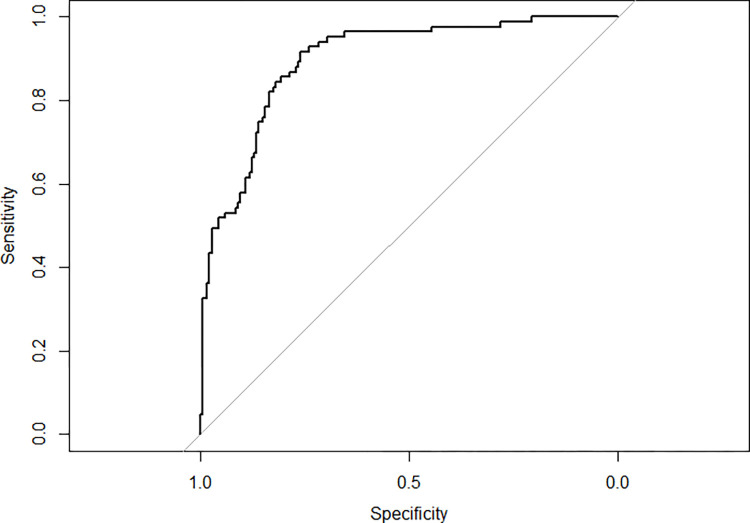
Receiver operating characteristic curve (basal model + physical function, physical role, and social function values).

A multivariate analysis of the 8 dimensions and SF-12 summary components, adjusted for age, sex, and the CVRF and comorbidities included in the previous model was subsequently performed. Only 3 dimensions were significantly associated in this final multiple regression model: physical function, physical role, and social function. A statistically significant inverse association was observed between the physical function and social function dimensions (p = 0.002 and p = 0.005) and an A multiple logistic regression model was constructed to evaluate the QoL in severe AS patients, adjusted for any variables that were significant in the baseline model inverse association close to significance (p = 0.052) was found between the physical role and severe AS (Table 3). The ROC curve showed high discriminative and predictive ability (ROC = 0.90 [0.86–0.90]) (Fig 2).

**Table 3:** Logistic regression model adjusted for statistically significant variables in the baseline model (CVRF and comorbidities) and values of ‘Physical functioning’, ‘Role physical’ and ‘Social functioning’.

Dimension	aOR (95%CI)^†^	P value
Physical functioning	0.94 (0.90–0.98)	**0.002** *
Role physical	0.89 (0.79–1.00)	0.052
Social functioning	0.95 (0.91–0.98)	**0.005** *

aOR, adjusted odds ratio; CI, confidence interval.

*p <0.05.

^**†**^ aOR (age, hemoglobin, smoking, hypercholesterolemia, low HDL-cholesterol, stroke, carotid stenosis, and chronic kidney failure)

## Discussion

There is a clear association between symptomatic severe AS and lower self-reported perception of QoL. In this study, patients with AS scored lower than controls in all QoL domains of the SF-12 questionnaire, also when adjusted for CVRF and comorbidities. In the final multiple logistic regression model, we observed a significant association between physical function and social function and AS and an almost statistically significant association with physical role after controlling for other CVRF and comorbidities.

Several tools are available for assessing QoL in the general population and in patients with specific chronic diseases. Disease-specific QoL scales focus on areas of life most affected by the disease and therefore have a higher sensitivity and specificity than general scales. However, the advantage of generic QoL instruments is that they can be used in different types of patients and populations and they can compare the relative impact of different diseases and obtain reference population values. Since no specific QoL scales for AS were available at the moment of initiation of the study, the generic SF-12 questionnaire was used in this study.

The SF-12 questionnaire measures general health and well-being and covers 8 domains: physical function, which addresses limitations in daily life due to health problems; physical role limitations due to physical problems; bodily pain, which evaluates the frequency of the pain and how it interferes on an individual level; overall health, which measures the individual’s perception of their general health status; vitality, which assesses energy and fatigue levels; social function, which measures the extent to which health status interferes with social activities; limitations in emotional role activities due to emotional problems; and mental health measured by psychological distress [14–16]. The results of this study show that severe AS affects all dimensions of patients’ QoL.

Published data on the impact of AS on patients’ QoL are scarce. In fact, only one study comparing the QoL of patients with symptomatic or asymptomatic severe AS with the general population in 3 age groups (41–60 years, 61–70 years, and > 70 years) has been conducted. This study found that QoL is lower in patients with symptomatic AS [17]. Our results are in line with these data and show that the QoL of patients ≥ 65 years with symptomatic severe AS is lower than that of controls. In contrast with van Geldorp *et al*., after adjusting for CVRF and comorbidities, only two SF-12 questionnaire dimensions were associated with AS (physical function and social function) and one, almost statistically significant, was considered clinically relevant (physical role). These associations could help identify QoL issues in patients who have few symptoms and, despite recommendation in current guidelines, are not referred for intervention.

A lower self-perceived QoL appears to have no correlation with disease severity. According to van Geldorp *et al*., there is no relationship between the degree of stenosis and the PCS and MCS scores of the SF-36v2 questionnaire. However, QoL does correlate with symptoms, since asymptomatic patients report a QoL similar to that of the general population [17]. Although everything points in this direction, it is not known whether the severity of symptoms is negatively correlated with self-perceived QoL, or what the specific weight of each symptom in the impact of AS on QoL might be. This is a question that should be investigated in prospective studies in the future.

In recent years, the study of QoL in patients with severe AS has focused on evaluating improvement after an intervention (AVR and/or TAVI), or on assessing the QoL of patients receiving palliative medical treatment in whom any intervention has been ruled out [17, 22–27]. HRQoL should also be taken into account when assessing health status and may be a useful parameter in the therapeutic decision-making process [10, 12, 28].

Current guidelines recommend earlier intervention in patients with severe AS, in both symptomatic and asymptomatic patients [7]. Compared to conservative treatment, surgical intervention improves prognostic of these patients. Despite these recommendations, between 30% and 60% of patients are still not referred for aortic valve replacement surgery [9]. Underestimation of the impact of aortic stenosis symptoms on quality of life may be one of the reasons of underestimation of surgical need in both types of patients. In this way, questionnaires such as SF-12 or EQ-5D may provide a quality of life measure that would complement the clinical data of patients and contribute to the improvement of therapeutic decisions [17, 29–32]. Given the differences observed in 3 of the SF-12 questionnaire dimensions (physical function, social function, and physical role), special attention to these items might mean that patients not otherwise considered for referral could be offered intervention [9].

This study has some limitations. Some cases who were potential candidates to participate may have died, which could mean that cases that were included had a better prognosis, bolstering the positive value of the results obtained. Likewise, controls may have been potential undiagnosed cases, since AS may not have clinical manifestations, even at advanced stages. This assumption would also further strengthen the results. With regard to sample size, 3 controls could not be selected for some cases, so the final sample size was slightly smaller than planned (221 instead of 261), thus decreasing the precision and statistical power. Despite matching by age, cases were older than controls due to the wide range used for matching (± 5 years), but age was taken into account in all regression models used. The effect of the other major CVRF and comorbidities on the QoL of cases and controls was taken into account in the regression models, but some residual confounding effect cannot be ruled out. The fact that the study was limited to people over 65 years could influence the magnitude of the association of QoL and severe AS. Finally, the results may not be applicable to other geographic areas.

## Conclusion

Individuals ≥ 65 years with symptomatic severe AS score worse in the 8 health dimensions and PCS and MCS of the SF-12 health questionnaire than paired controls. In the multiple logistic regression models, all SF-12 dimensions and summary components are inversely associated with symptomatic severe AS in people ≥ 65 years. In the final regression model, an inverse association between the physical function and social function dimensions of the SF-12 questionnaire is observed in severe AS. In all cases, the discriminative and predictive ability of the association of the SF-12 questionnaire dimensions with severe AS was good. The use of quality-of-life scales provides evidence for patient‐centered care and could improve the management of severe AS, reduce delayed referral and improve patients’ QoL.
